# An Interesting Case of Treatment-Resistant Ventricular Tachycardia Secondary to Pheochromocytoma and Left Ventricular Non-compaction

**DOI:** 10.7759/cureus.25483

**Published:** 2022-05-30

**Authors:** Zahid Khan

**Affiliations:** 1 Cardiology and General Medicine, Barking, Havering and Redbridge University Hospitals NHS Trust, London, GBR; 2 Cardiology, Royal Free Hospital, London, GBR

**Keywords:** implantable cardiac defibrillator (icd), airway intubation, normal coronary angiogram, direct current cardioversion, amiodarone and lidocaine, left ventricular non compaction, alpha channel blockers, beta adrenergic blockers, ventricular tachycardia (vt) storm, adrenal pheochromocytoma

## Abstract

A 51-year-old patient was admitted with chest pain and broad complex ventricular tachycardia. He received three consecutive direct cardioversion (DC) shocks and was commenced on amiodarone infusion via a central venous catheter or central line (CVC). He responded to treatment and normal sinus rhythm (NSR) was achieved. He had elevated troponin I and underwent coronary angiogram which initially was thought to be responsible for his ventricular tachycardia. Coronary angiogram (CAG) showed unobstructed coronary arteries. He was recently diagnosed with pheochromocytoma and was commenced on Phenoxybenzamine 10 mg two months back. He developed ventricular tachycardia (VT) again the next day that did not respond to four consecutive direct cardioversion shocks (DC) and antiarrhythmic medications. He was intubated and ventilated to terminate his VT and was transferred to the intensive care unit (ICU). He remained intubated for 48 hours and he remained in NSR, after which he was extubated. He was commenced on bisoprolol and was later stepped down to the coronary care unit (CCU). Cardiac magnetic resonance imaging (CMR) showed left ventricular non-compaction (LVNC) or possibly myocarditis in view of patient's known history of pheochromocytoma. He was discussed with surgical team at another hospital for surgical resection of the adrenal tumor and had a few further runs of VT while he was waiting to be transferred. The patient finally underwent surgical resection of the tumor and was booked for implantable cardioverter defibrillator (ICD) in view of his VT. This was an interesting case of treatment-resistant VT driven by pheochromocytoma and LVNC, and it is important to be familiar with the fact that conventional therapies may fail in these patients and may require intubation and ventilation to terminate VT storms.

## Introduction

Pheochromocytoma is a catecholamine-producing tumor originating from the adrenal medulla or extra-adrenal and typically presents with a triad of abrupt episodes of headache, palpitations, and profuse sweating which may be accompanied by either labile or sustained hypertension [1,2]. Pheochromocytomas can also arise extra-adrenal chromaffin cells and produce hormones such as catecholamines, epinephrine and norepinephrine and the cardiovascular effects are determined by the type of hormone released into the circulation by the tumor [3]. The most common catecholamine-induced tachyarrhythmias caused by pheochromocytoma include severe and refractory sinus tachycardia, atrial fibrillation, and ventricular tachycardia which mainly arise due to catecholamine excess [3].

Tumors arising from the chromaffin cells of the adrenal medulla and extra adrenal sympathetic paraganglia that produce excess epinephrine and norepinephrine are responsible for the life threatening arrhythmias and labile blood pressure [3]. The most common arrhythmia in patients with pheochromocytoma is sinus tachycardia followed by atrial fibrillation (60%), bradyarrhythmias (20%) and ventricular tachycardia (13%) and a smaller percentage have unspecified supraventricular tachycardias [4]. Majority pheochromocytoma patients suffer from episodic/ non-persistent hypertension and they are at risk of developing heart failure or myocardial infarction [4].

Pheochromocytoma can occasionally also present with severe left ventricular systolic dysfunction (LVSD) and can present with stress cardiomyopathy that may mimic takotsubo cardiomyopathy [5]. In this case report, we present a case of 51-year-old patient who presented with chest pain secondary to ventricular arrhythmia caused by pheochromocytoma.

## Case presentation

A 51-year-old patient presented to the accident and emergency (A and E) department with central crushing chest pain and palpitations. On arrival, the patient was found to be in broad complex ventricular tachycardia and received three consecutive direct current cardioversion (DCCV) shocks followed by amiodarone infusion. The patient reverted to normal sinus rhythm and was admitted to coronary care unit (CCU). His past medical history (PMH) includes type 2 diabetes mellitus (T2DM), hypercholesterolemia, sciatica, psoriasis and psoriatic arthritis, childhood tuberculosis, and a recent diagnosis of pheochromocytoma. His regular medications include metformin, atorvastatin, phenoxybenzamine 10 mg OD, calcipotriol, lansoprazole, and co-codamol. He had a history of smoking 45-pack-year and was a non-drinker. He was on secukinumab for psoriasis which was on hold due to his recent diagnosis with pheochromocytoma based on computerized tomography scan of chest, abdomen, and pelvis (CT CAP) for right upper quadrant (RUQ) pain and his background history of immunosuppression and enlarged parotid gland.

His vital signs on arrival were as follows: heart rate (HR) was 192 beats per minute (bpm), blood pressure (BP) (standing) was 116/76 mmHg and BP (supine) was 111/70 mmHg, and SpO_2_ was 97%. His laboratory tests showed elevated troponin (T) 9 ng/L and repeat troponin was 143 ng/L. His electrocardiogram (ECG) showed broad complex ventricular tachycardia. He underwent coronary angiogram the following day that showed unobstructed coronary arteries. His metanephrine and normetanephrine levels were 12132 pg/mL and 3960 pg/mL. Other laboratory results are shown in Table 1.

**Table 1:** The trend of laboratory results for this patient during hospital admission.

Blood test	Day 1	Day 3	Day 7	Normal value
Hemoglobin	123	132	127	133-173 g/L
White cell count	15	12.3	11.6	3.8-11 × 10^9^/L
Neutrophil	12	11.2	9.8	2-7.5 × 10^9^/L
C-reactive protein	23	25	20	0-5 mg/L
Urea	6.5	7.2	7.3	2.5-7.8 mmol/L
Creatinine	81	91	93	59-104 μmol
Sodium	139	140	136	133-146 mmol/L
Potassium	4.9	5.2	4.9	3.5-5.3 mmol/L
Metanephrine	12132	Not checked	Not checked	12-60 pg/mL
Normetanephrine	3960	Not checked	Not checked	18-111 pg/mL
Magnesium	0.91	0.95	0.93	0.65-1.05 mmol/L

**Figure 1:**
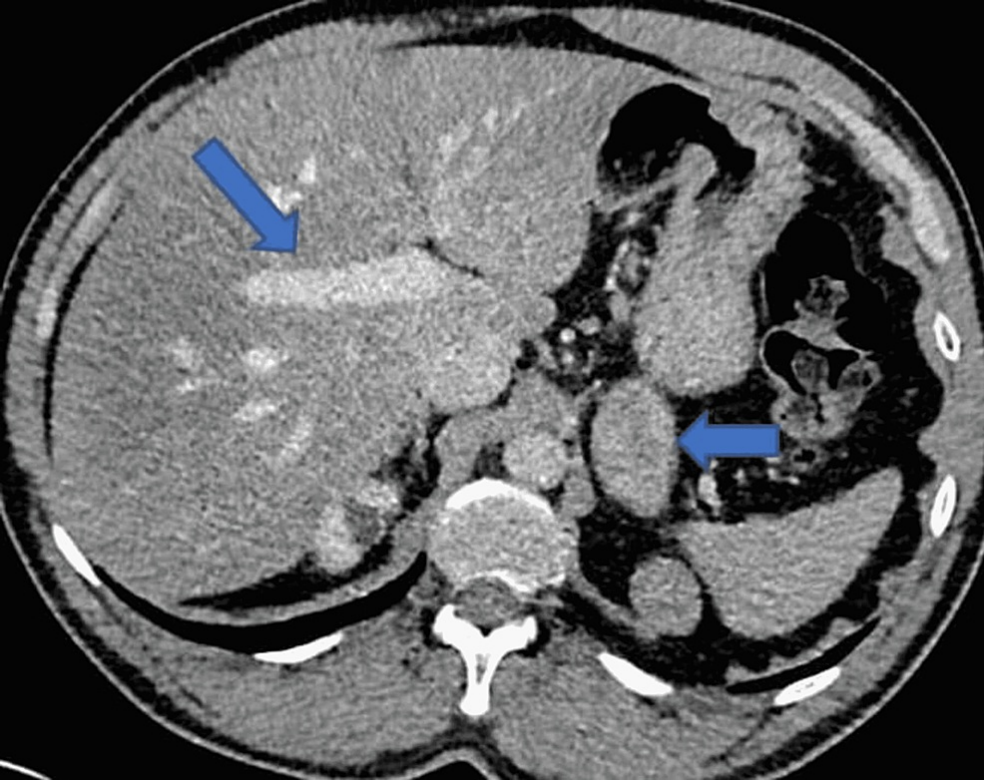
Computerized tomography scan of chest, abdomen, and pelvis shows hepatic steatosis (arrow on the right) and left adrenal tumor (arrow on the left).

**Figure 2:**
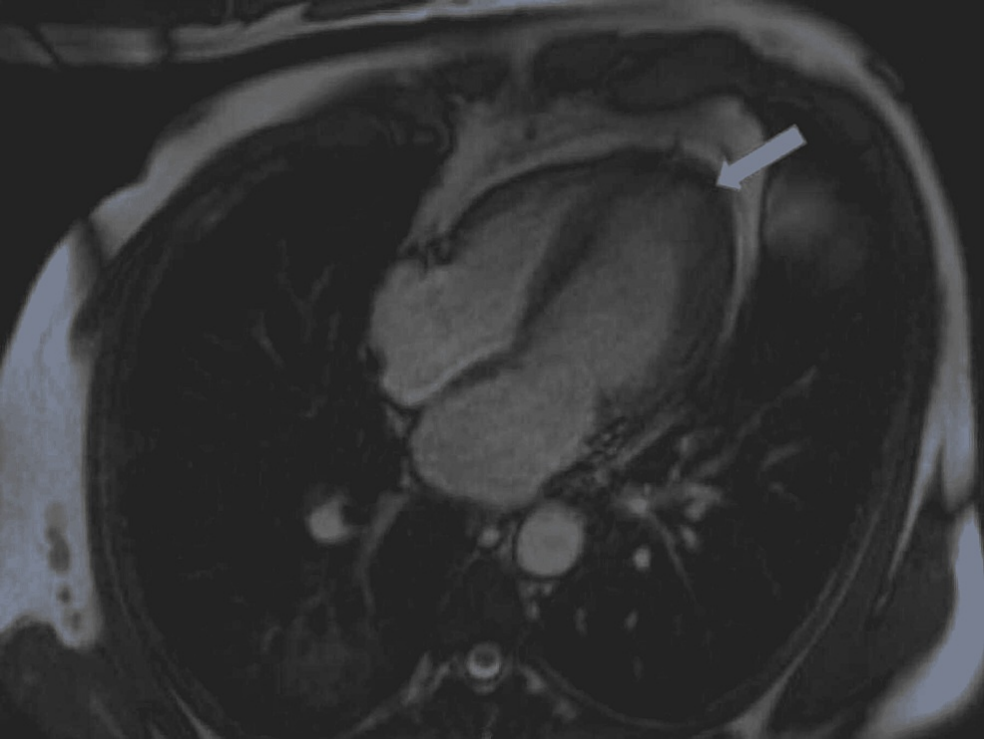
Cardiac magnetic resonance (CMR) shows left ventricular non-compaction; dark area around the left ventricular cavity (arrow)

**Figure 3:**
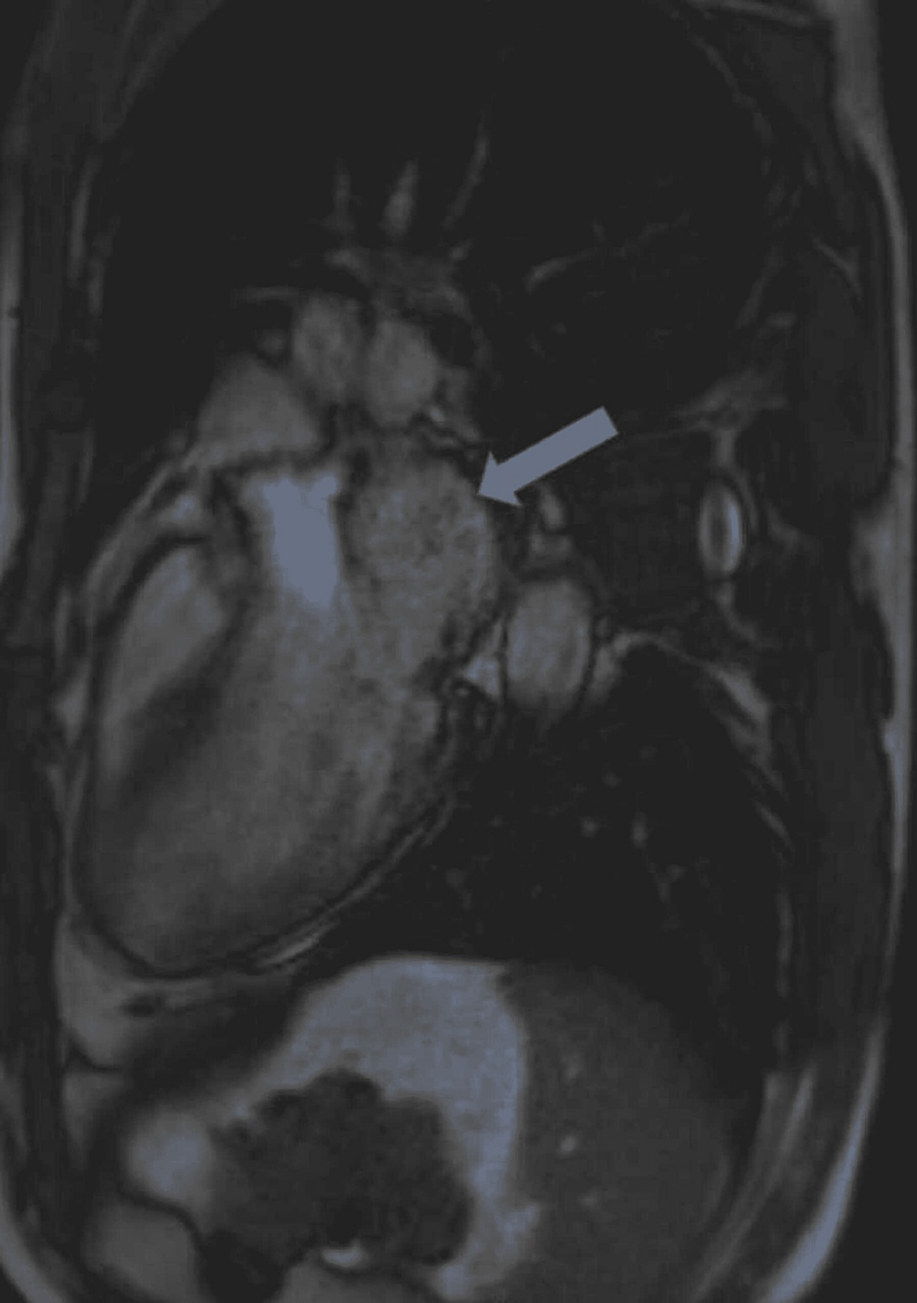
Cardiac magnetic resonance imaging shows left ventricular non-compaction cardiomyopathy (arrow).

He developed broad complex regular ventricular tachycardia (VT) the next day again and was delivered 4 DCCV shocks, and the patient did not respond to them. He was given further amiodarone 300 mg bolus followed by lidocaine 50 mg and then 100 mg boluses; however, the patient remained in VT storm. He was intubated due to VT storm and was transferred to intensive care unit (ICU) and was commenced propofol along with inotropic support in view of hypotension. The patient remained in normal sinus rhythm (NSR) and was extubated after 48 hours. He remained hypotensive and was not tolerating even small doses of metoprolol. He was then commenced on metoprolol 12.5 mg TDS after his BP improved which he tolerated quite well. He had further run of VT after being stepped down to CCU. He had a CT CAP which showed 5 cm left adrenal pheochromocytoma (Figure 1). He underwent cardiac magnetic resonance imaging (CMR) that showed high normal left ventricular (LV) cavity when indexed for body surface area (BSA) with normal LV mass (Figures 2, 3).

Preserved systolic function with left ventricular ejection fraction (LVEF) was 58%. The imaging findings revealed small right ventricular (RV) cavity with normal wall thickness and systolic function, tricuspid annular plane systolic excursion (TAPSE) 18 mm, right ventricular ejection fraction (RVEF) of 74%. There was extensive, almost circumferential sub-epicardial late gadolinium enhancement predominantly involving the basal to mid anterior, lateral, and inferior walls. There was also an additional intramyocardial late gadolinium enhancement in the basal to mid septum extending to the inferior RV/LV insertion point which was in keeping with left ventricular non-compaction (LVNC) (Tables 2, 3).

**Table 2:** Cardiac magnetic resonance imaging findings of both left and right ventricular functions. EDV: end-diastolic volume; ESV: end-systolic volume; EF: ejection fraction; LV: left ventricle; RV: right ventricle

Chamber	EDV (mL)	ESV (mL)	SV (mL)	EF (%)	Mass (g)
LV	195	81	114	58	159
	119-203	33-77	78-134	57-75	107-187
RV	94	24	70	74	-
	119-219	32-92	73-141	50-78	40-96

**Table 3:** Cardiac magnetic resonance imaging indexed systolic and diastolic findings. EDVi: end-diastolic volume index; ESVi: end-systolic volume index, SVi: systolic volume index; mass-i: mass index; LV: left ventricle; RV: right ventricle

Chamber	EDVi (mL/m^2^)	ESVi (mL/m^2^)	SVi (mL/m^2^)	Mass-i (g/m^2^)
LV	96	40	56	79
	64-100	17-39	43-67	57-91
RV	47	12	35	-
	63-111	18-46	39-71	21-49

The patient had further episodes of VT and was transferred to a specialist arrhythmia and endocrine center; he underwent successful surgical resection of the tumor followed by dual chamber implantable cardioverter defibrillator (ICD) implantation. ICD checks were satisfactory, and the patient was discharged home with outpatient follow-up. Repeat ICD checks did not reveal any further VT episodes and the patient remained symptom-free.

Histology from the biopsies showed a cellular tumor (Pheochromocytoma of the Adrenal gland Scaled Score {PASS} +2) of the adrenal medulla composed of large polygonal cells with abundant basophilic cytoplasm and markedly pleomorphic nuclei (PASS +2). Cells were predominantly arranged in a nested pattern. There was no spindling of the tumor cells, nor lymphovascular permeation is identified. Capsular invasion is seen (A5, PASS +1) and the tumor extends into the adjacent periadrenal fibromuscular and adipose tissue (PASS +2). Adrenal cortex is unremarkable and features are consistent with pheochromocytoma.

## Discussion

Pheochromocytoma and LVNC are both rare diseases and very rarely occur together [3,5]. Spongy myocardium is another term used for LVNC and it is a rare abnormality of the left ventricular myocardium consisting of two layers; compacted and non-compacted layers.

Pheochromocytoma can present with life-threatening arrhythmia that may be resistant to standard treatment and it can also present with cardiomyopathy [6]. The diagnosis of pheochromocytoma can be challenging particularly in young patients due to the varied presentation [7]. The LVNC is a rare subtype of cardiomyopathy, characterized by predominant left ventricular trabeculations and thinning of the epicardium associated with a high risk of heart failure (HF), thromboembolism, arrhythmia, and sudden cardiac death. Pheochromocytoma has been reported to be associated with stress-induced cardiomyopathy or takotsubo cardiomyopathy with characteristics ballooning of the apical, anterior, and inferior cardiac walls [8,9]. Other studies have reported the association between pheochromocytoma, transient myocardial dysfunction, and acute coronary syndrome (ACS) caused by excess catecholamine release [10-12].

Previous research has reported that patients presenting with acute myocardial infarction secondary to pheochromocytoma crises had significant coronary arteries atherosclerosis and it’s likely that excess catecholamine release leads to hemodynamic compromise of the myocardium accelerating myocardial cell death. This can also be explained by severe coronary arteries spasm caused by excessive catecholamine release from the primary adrenal tumor [13]. A case report of a 65-year-old patient presenting with chest pain and initial coronary angiogram showed 90% left anterior descending artery (LAD) stenosis and had successful primary percutaneous coronary intervention (PPCI) to the LAD. He represented to the hospital five days after discharge with markedly fluctuant BP of 224/76 and 70/55 mmHg, chest pain and VT, and ECG showed T-wave inversion (TWI) V1-V4. His CT scan showed adrenal tumor and after successful surgical removal of the tumor; his blood pressure improved, and ECG changes resolved completely [13].

Another study based on 26 pheochromocytoma patients reported normal echocardiographic evaluations in 62.1% of the patients, 27.6% patients had concentric LV hypertrophy with normal LV systolic function (LVSF) and 10.3% had LVSD [14]. Only one symptomatic patient from this study had catecholamine cardiomyopathy with transient LVSD [2]. Catecholamines can cause myocardial necrosis, focal myofibrillar degeneration, and fibrous scar formation which can result in LVSD resembling takotsubo cardiomyopathy and long-term elevated catecholamine levels can lead to myocardial hypertrophy and heart failure [3].

**Figure 4:**
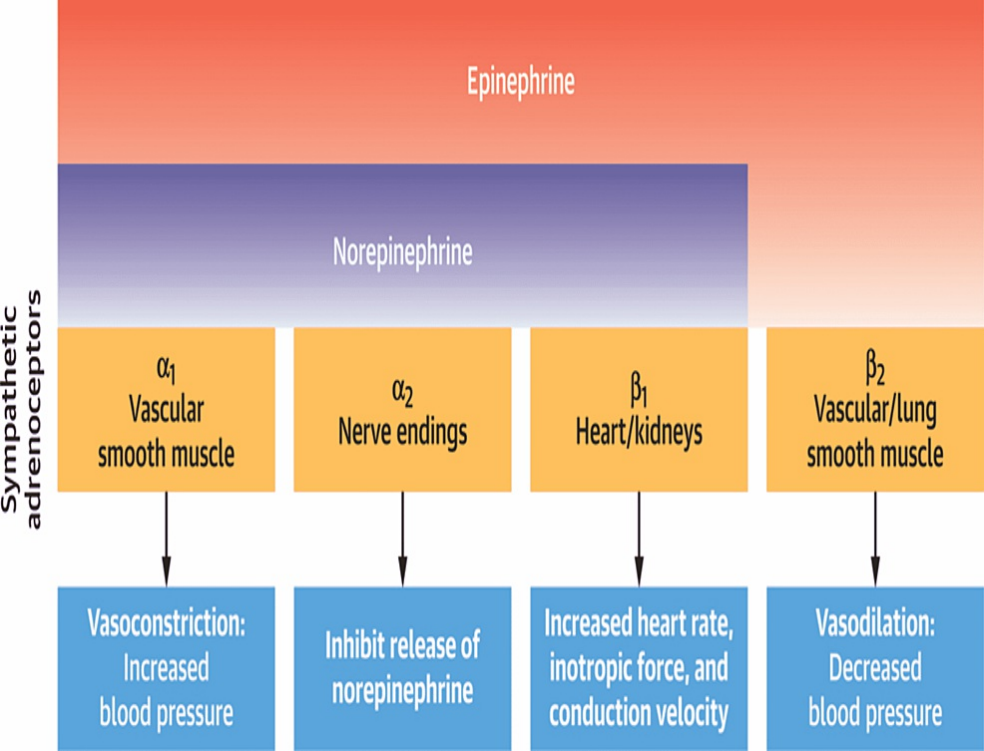
Catecholamine binding affinities and adrenoceptor actions. The image is obtained with permission from the corresponding author (Karel Pacak) [16].

**Figure 5:**
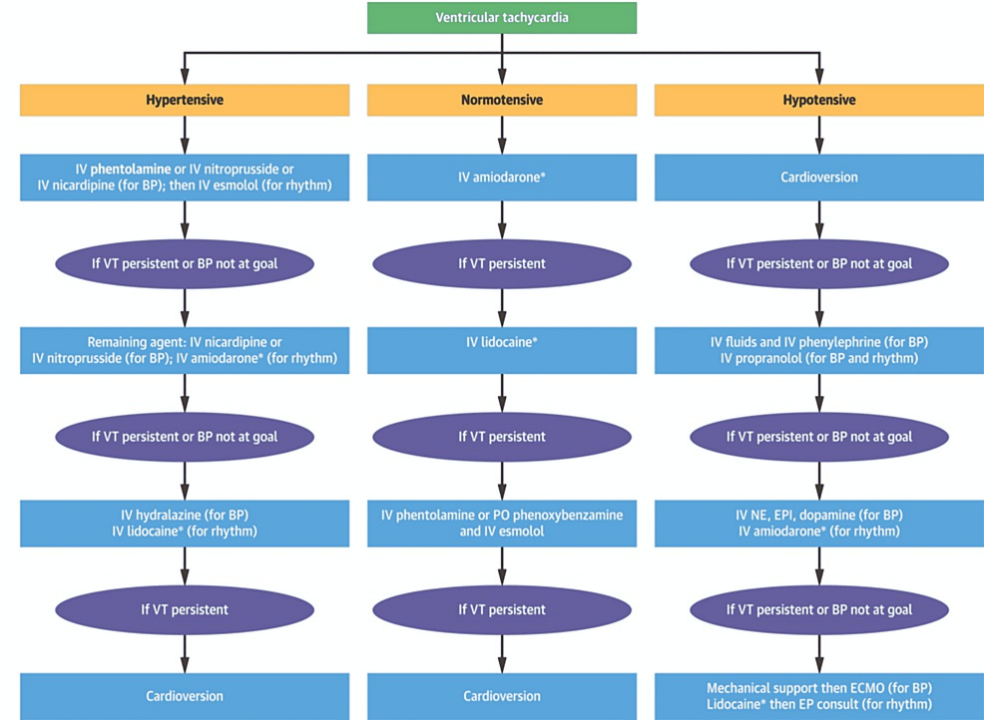
Management of catecholamine-induced tachyarrhythmias. The image is obtained with permission from the corresponding author (Karel Pacak) [16].

Cardiovascular manifestations from pheochromocytoma such as arrhythmias, and BP abnormalities account for 71% of the mortality and 90% of these patients have hypertension with 75% of patients having paroxysm of hypertension at least once a week [14,15]. A retrospective cohort study from 2004 to 2019 based on 650 patients showed that 10.9% of patients experienced tachyarrhythmia despite being on beta-blocking therapy. From this cohort of patients, 98.6% had experienced sinus tachycardia (ST) once at least, 11.3% atrial fibrillation, 5.6% atrial flutter; and 4.2% VT [13]. Management of catecholamine-induced tachyarrhythmias is quite complex as they may not respond to conventional therapy mostly and patients can deteriorate rapidly, and this is made more challenging by the BP alteration in patients with catecholamine excess. This can lead to serious complications such as hypertensive crises, hypotensive shock, cardiac arrest, and death [14-16]. Very rarely patients with pheochromocytoma may present with hemorrhagic necrosis of the tumor [17]. The management of pheochromocytoma-induced VT is presented in Figures 4, 5.

A systemic review based on 163 cases from 150 articles reported that 63 patients had dilated cardiomyopathy, 38 had takotsubo cardiomyopathy, 30 inverted takotsubo cardiomyopathy, 10 hypertrophic obstructive cardiomyopathy (HOCM), eight had myocarditis, and 14 patients had unspecified cardiomyopathy. A total of 65% of patients lacked the classic association of hypertension with pheochromocytoma and only 4% of patients had the triad of headache, palpitations, and diaphoresis. Also, 96% of patients showed improvement in their symptoms after resection of pheochromocytoma while lack of resection was associated with death or cardiac transplantation in 44% of patients [18].

## Conclusions

Pheochromocytoma and left ventricular non-compaction are two rare diseases and occasionally can be found together. Pheochromocytoma-induced VT can be difficult to manage due to excess catecholamine release. The patient in our study had both conditions and presented with ventricular tachycardia that initially responded to direct current cardioversion but subsequently stopped responding to pharmacological therapy or direct current cardioversion and the patient was eventually intubated and ventilated to break the ventricular tachycardia storm. Physicians need to be aware of the management of this condition as they may not respond to traditional treatment and if left alone, patients can deteriorate rapidly resulting in catastrophic outcomes. This patient had resection of the tumor and has been doing well since.
